# Misregulated E-Cadherin Expression Associated with an Aggressive Brain Tumor Phenotype

**DOI:** 10.1371/journal.pone.0013665

**Published:** 2010-10-27

**Authors:** Laura J. Lewis-Tuffin, Fausto Rodriguez, Caterina Giannini, Bernd Scheithauer, Brian M. Necela, Jann N. Sarkaria, Panos Z. Anastasiadis

**Affiliations:** 1 Department of Cancer Cell Biology, Mayo Clinic, Jacksonville, Florida, United States of America; 2 Department of Pathology, Mayo Clinic, Rochester, Minnesota, United States of America; 3 Department of Radiation Oncology, Mayo Clinic, Rochester, Minnesota, United States of America; The Beatson Institute for Cancer Research, United Kingdom

## Abstract

**Background:**

Cadherins are essential components of the adherens junction complexes that mediate cell-cell adhesion and regulate cell motility. During tissue morphogenesis, changes in cadherin expression (known as cadherin switching) are a common mechanism for altering cell fate. Cadherin switching is also common during epithelial tumor progression, where it is thought to promote tumor invasion and metastasis. E-cadherin is the predominant cadherin expressed in epithelial tissues, but its expression is very limited in normal brain.

**Methodology/Principal Findings:**

We identified E-cadherin expression in a retrospective series of glioblastomas exhibiting epithelial or pseudoepithelial differentiation. Unlike in epithelial tissues, E-cadherin expression in gliomas correlated with an unfavorable clinical outcome. Western blotting of two panels of human GBM cell lines propagated either as xenografts in nude mice or grown under conventional cell culture conditions confirmed that E-cadherin expression is rare. However, a small number of xenograft lines did express E-cadherin, its expression correlating with increased invasiveness when the cells were implanted orthotopically in mouse brain. In the conventionally cultured SF767 glioma cell line, E-cadherin expression was localized throughout the plasma membrane rather than being restricted to areas of cell-cell contact. ShRNA knockdown of E-cadherin in these cells resulted in decreased proliferation and migration *in vitro*.

**Conclusions/Significance:**

Our data shows an unexpected correlation between the abnormal expression of E-cadherin in a subset of GBM tumor cells and the growth and migration of this aggressive brain tumor subtype.

## Introduction

Glioblastoma multiforme or glioblastoma (GBM) continues to be the most frequently diagnosed and lethal of primary brain tumors. This WHO grade IV brain tumor is remarkable in both its morphological diversity and biologic aggressiveness, the latter being partly due to its diffusely infiltrative nature [1], [2]. Extensive, diffuse parenchymal invasion is an important reason for failure of the most accepted treatment modalities, including surgical resection combined with radiation and chemotherapy. These methods effectively target the main tumor and any remaining tumor cells that are proliferating. However, non-proliferating tumor cells are more resistant to cytotoxic therapies, and migration away from the grossly detectable tumor mass can lead to localized treatment failure beyond the surgical resection bed and high-dose radiation zone [3].

Migrating cells undergo changes in cell-cell and cell-extracellular matrix (ECM) adhesion that facilitate detachment from their surroundings and promote motility. The transmembrane protein family of cadherins regulates cell-cell adhesion during a variety of biological processes, including tissue morphogenesis as well as tumor invasion/metastasis [4]–[7]. Classical cadherins mediate cell-cell adhesion and induce adhesion-related signaling via their interaction with p120 catenin (p120) and β-catenin [8]–[10]. These catenins, in turn, regulate a variety of processes, including cadherin clustering and stabilization, modulation of Rho GTPase signaling, actin cytoskeleton rearrangement, and gene transcription [11], [12]. Ultimately, such regulation governs the balance between cell-cell adhesion on the one hand and cell motility on the other, placing the cadherin-catenin complex in a significant position to regulate both tumor cell proliferation and invasion.

Cadherins are critical players in an important mechanism that underlies both developmental processes and epithelial tumorigenesis: the form of metaplasia known as epithelial-to-mesenchymal transition (EMT). During EMT, the expression of the epithelial E-cadherin is reduced in exchange for increased expression of mesenchymal cadherins such as N-cadherin or cadherin-11 [13]. One result of this transition is cells that are more motile. A developmental example of EMT underlies the formation of the neural tube, allowing N-cadherin expressing cells in the developing neuroepithelium to separate and migrate away from surrounding E-cadherin expressing cells [14], [15]. Further rounds of differential cadherin expression are also thought to promote regionalization during later stages of nervous system development [16], [17]. The classical cadherin superfamily (both Types I and II) consists of more than 18 members, the differential expression of which occurs at all stages of nervous system development and persists into adulthood. Neurons that express a particular cadherin preferentially form synapses with, and migrate into regions that express the same cadherin [17], [18]. Thus differential cadherin expression is a general mechanism for regulating cell motility and promoting brain regionalization. During epithelial tumor progression this normal morphogenetic process is hijacked, and EMT is thought to induce the invasion and metastasis of, among others, breast, kidney, and colorectal cancer cells [6], [15], [19].

Previous examinations of cadherin expression in the normal human adult nervous system indicate that E-cadherin expression is rare, and limited to the arachnoid membrane [20]–[25]. In mice, E-cadherin is also expressed in neural stem cells, where it regulates self renewal [26]. On the other hand, mesenchymal cadherins such as N-cadherin or cadherin 11 are expressed in multiple brain regions, including the cortex and hippocampus, and are important to normal nervous system function [18], with a possible role in gliomagenesis or invasiveness. Only a handful of studies have focused on the involvement of cadherins or catenins in high-grade adult gliomas (anaplastic astrocytomas or GBMs) [22]–[25], [27]–[29] (reviewed in [30]). Most of these studies examined resected tumors or glioblastoma cell lines and focused on the expression of E-cadherin and N-cadherin protein. Consistently, E-cadherin protein expression was found to be rare to non-existent in both tumor and normal brain tissue [20], [22], [24], [25], [31], while N-cadherin was extensively represented. In contrast to these results, two recent studies argue that E-cadherin expression decreases with brain tumor grade when compared to normal brain [27], [32], raising the possibility that a classic EMT is involved in glioma progression. On balance, the existing data are inconclusive as to the pathobiologic role of E-cadherin in high-grade adult gliomas.

We show here that E-cadherin expression is very limited in normal brain, and rare in astrocytic and GBM tumors. Previously we had examined the histopathologic, immunophenotypic, and molecular characteristics of a rare form of GBM exhibiting varying degrees of epithelial or pseudo-epithelial differentiation [33]. In this study we found E-cadherin protein to be expressed in one third of these tumors, and this expression correlated with worse patient outcome. Therefore we sought to determine if the expression of E-cadherin had functional consequences related to tumor biology. Using multiple cell line models of GBM, it was determined that expression of E-cadherin correlated with growth, migration, and invasiveness. Taken together, our data suggest a novel and potentially important role for E-cadherin in the lethality of this subset of high-grade adult gliomas.

## Results

### E-cadherin expression is rare in GBM

We used immunohistochemistry to examine E-cadherin expression in brain tumor and non-neoplastic brain samples from three tissue microarrays (TMAs): two constructed at the Mayo Clinic containing a total of 83 glioblastoma (predominantly) or anaplastic astrocytoma samples and one glioma invasion tissue microarray containing 31 matched brain tumor rim/core samples (a generous gift from M. Berens [34]). We initially probed the TMAs with the clone 36 E-cadherin antibody, which is highly specific for E-cadherin when used with Western Blot. However, in the context of the formalin-fixed, paraffin-embedded samples of the TMAs, this antibody produced non-specific staining (Figure S1), similar to what has been reported previously [35]. Therefore the 4A2C7 antibody clone, which does not have this problem (Figure S1) was used to evaluate E-cadherin expression. While E-cadherin staining was clearly present in the infiltrating ductal breast carcinoma samples on these TMAs, we did not find any E-cadherin expression in any brain tumor or non-neoplastic brain sample (data not shown). Thus brain- and brain tumor-associated E-cadherin expression is rare.

### E-cadherin expression in GBM with epithelial/pseudoepithelial differentiation

**Figure 1:**
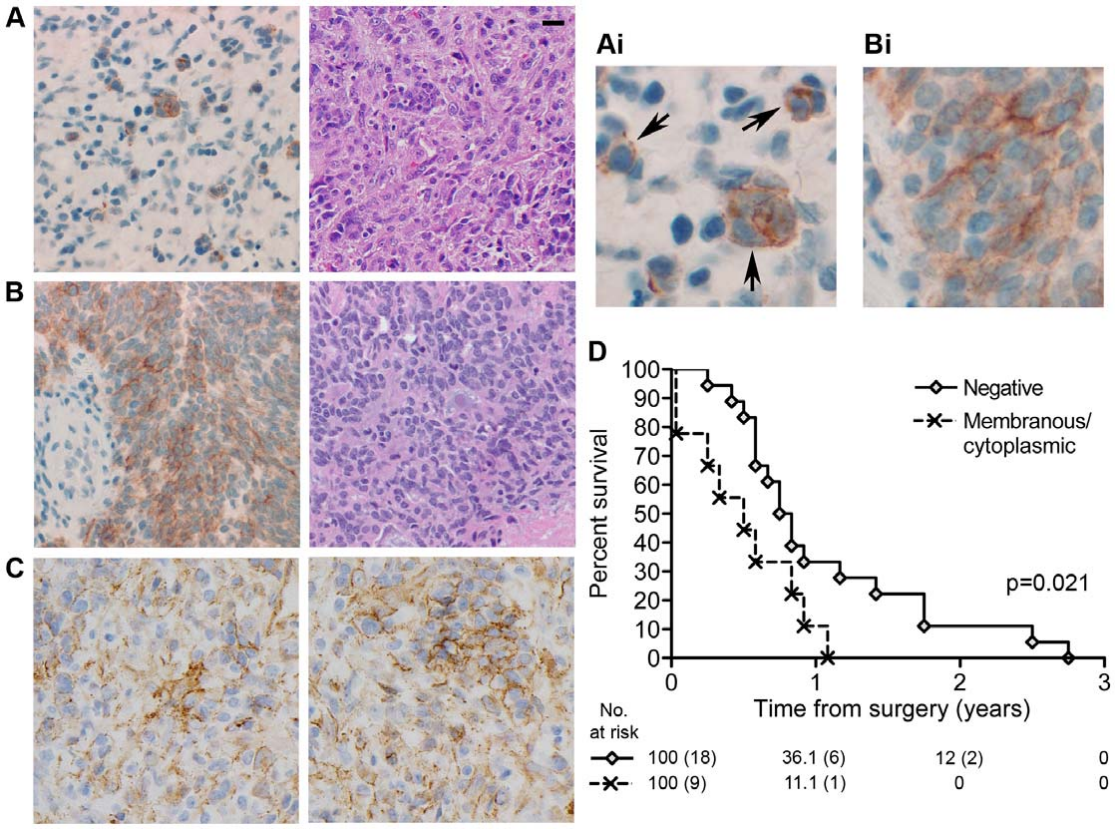
E-cadherin expression correlates with worse outcome for patients with glioblastomas exhibiting epithelial appearance. A. E-cadherin protein in 8 of the 9 positive cases was focally restricted to discrete nests of tumor cells, reflecting areas of epithelial-like differentiation. In these positive cells E-cadherin was found primarily on the plasma membrane. However, unlike in normal epithelial cells, the localization of E-cadherin was not concentrated at areas of homotypic cell-cell contact, but rather localized uniformly along the plasma membrane (indicated by arrows in Ai). Left: Immunostain for E-cadherin (magnified in Ai to show detail); Right: H&E. The scale bar is 20 µm and applies to all Figure 1 images except Ai and Bi. B. 1 of the 9 E-cadherin positive cases showed an unusually high expression of E-cadherin which was located both on the membrane and cytoplasmically. Left: Immunostain for E-cadherin (magnified in Bi to show detail); Right: H&E of the tumor's epithelial component. C. Two independent examples of β-catenin localization by immunostain. Unlike E-cadherin, β-catenin is distributed throughout the tumor samples. D. Individual cases of glioblastoma exhibiting epithelial or pseudoepithelial differentiation were analyzed by immunohistochemistry for the absence (Negative) or presence (Membraneous/cytoplasmic) of E-cadherin protein. This data was correlated with overall survival using Kaplan-Meier analysis. There is a statistically significant survival difference (p = 0.021).

We also performed immunohistochemistry to analyze E-cadherin protein expression in tissue samples from 27 individual cases with adequate clinical follow up of a rare sub-type of GBM with epithelial/pseudoepithelial differentiation. These tumors likely represent primary GBM based on a relatively short duration of symptoms in patients with available history and lack of a documented lower grade precursor per established criteria [36]. In addition, molecular genetic changes typical of primary GBM were present, including *EGFR* amplification (25%) or gain of chromosome 7 without amplification (50%), and whole chromosome 10 loss (57%). E-cadherin expression was present in 9 cases (33%). The majority of the E-cadherin positive cases (8 out of 9 cases) exhibited a restricted pattern of E-cadherin immunostaining, in which E-cadherin was focally expressed in discrete nests of tumor cells, usually reflecting areas of epithelial-like differentiation (Figure 1A, Ai). In these positive cells, E-cadherin was expressed primarily on the plasma membrane, but was dispersed, rather than concentrated at areas of homotypic cell-cell contact as would be expected (see [37] for an example of normal E-cadherin distribution). In a single case (Figure 1B, Bi) E-cadherin expression was more diffusely spread throughout most cells in the epithelial component of the tumor. In this tumor, E-cadherin immunostaining was both membranous and cytoplasmic. In contrast to E-cadherin expression, β-catenin was present in all cases studied (Figure 1C), with a membranous and cytoplasmic (56%) or membranous only (44%) staining pattern. Patients whose tumors expressed E-cadherin demonstrated poorer overall survival compared to those that did not (Figure 1D; statistically significant at p = 0.021, log rank test). Although E-cadherin immunostaining was more frequent in GBM with epithelial-like differentiation (58%) compared with the adenoid (A-GBM) and epithelioid (E-GBM) histologic subgroups (13%) [33], only E-cadherin expression was associated with survival. In contrast, there were no significant associations between survival and age, tumor location, tumor size, extent of resection, β-catenin immunostaining, molecular cytogenetic abnormalities, or proliferative indices, nor between E-cadherin immunostaining and any of those parameters in this patient population (p>0.05).

### E-cadherin expression in the GBM xenograft model

**Figure 2:**
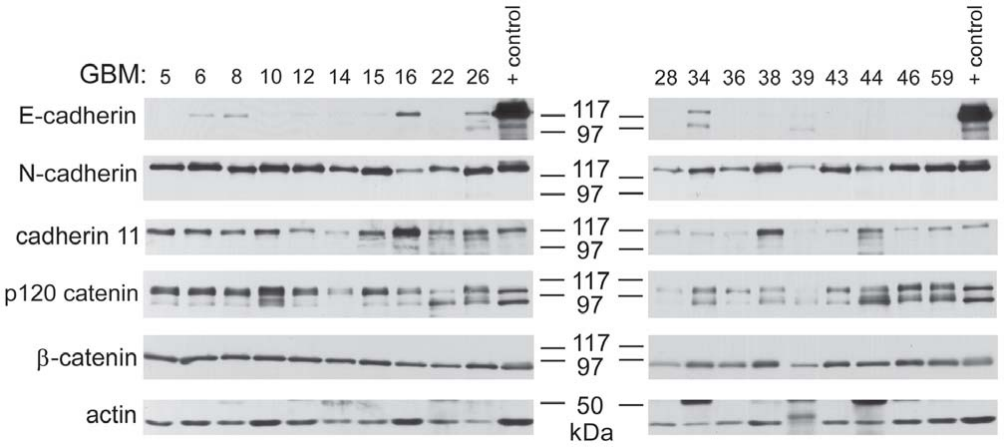
E-cadherin protein expression occurs in a subset of xenograft glioma lines. Nineteen glioma cell lines propagated as xenografts in mouse flank were examined by Western Blot for expression of various cadherin and catenin proteins. Actin serves as a loading control. Positive control lysates are from MCF7 cells (E-cadherin, β-catenin), UMRC3 cells (N-cadherin), and MDA231 cells (cadherin-11). Low levels of E-cadherin expression were detected in a small subset of these glioma xenografts.

Because the expression of E-cadherin in normal central nervous system tissue is rare and correlated with worse outcome for patients with GBM, we used an experimental model of GBM to follow up this observation. The Mayo GBM xenograft model is comprised of 19 primary GBM xenografts that were established directly from patient tumor specimens and passaged exclusively *in viv*o as flank xenografts. Orthotopic implantation of short-term explant cultures derived from these xenograft lines results in tumors with morphologic and genetic features similar to those seen in the primary patient specimens. Importantly, this GBM model frequently recapitulates several key features of the human disease that are lost in conventionally-cultured glioma cell lines, including retention of markers such as EGFR overexpression and a tendency for the orthotopically-implanted cells to invade throughout the mouse brain [38]–[40]. Whole cell lysates were made from 19 of these tumor lines using flank tumor specimens. These lysates were analyzed by Western blot for the expression of various human cadherins and catenins (Figure 2). All of the lines expressed β-catenin and p120 catenin, both important components of the cadherin/catenin cell-cell adhesion signaling complex. Most of the lines expressed N-cadherin and/or cadherin-11, which are frequently found in brain tumors and normal brain. Surprisingly, 5 of the 19 lines examined (GBMs 6, 8, 16, 26, and 34) expressed substantial amounts of E-cadherin. The relative amount of E-cadherin protein expression was low in the GBM xenograft lines compared to its expression in the E-cadherin positive MCF7 breast cancer cell line, which was used in this and later experiments as an example of a typical epithelial cell. The difference in E-cadherin protein levels among these cell lines was also reflected in E-cadherin mRNA levels (Lewis-Tuffin, data not shown) and may be due to a focal rather than general expression of E-cadherin in these tumors (similar to Figure 1A).

**Figure 3:**
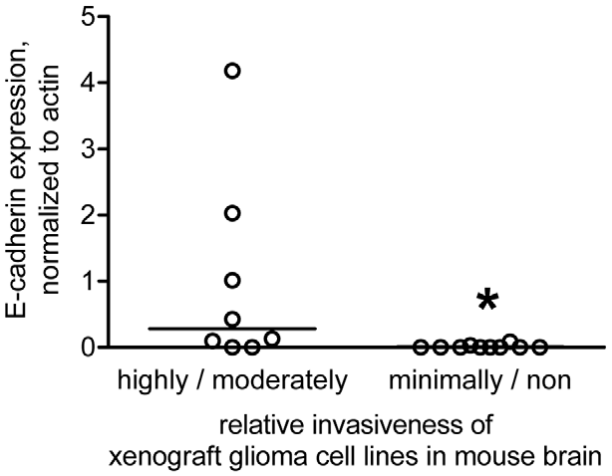
E-cadherin protein expression correlates with glioma cell invasiveness in an orthotopic xenograft mouse model. Xenograft cell lines 5, 6, 8, 10, 12, 14, 15, 16, 22, 26, 28, 34, 36, 38, 43, 44, 46, and 59 were examined for relative invasiveness following orthotopic injection into mouse brain. Lines were categorized as highly, moderately, minimally, or non invasive. The level of E-cadherin expression (relative to actin expression) determined by Western blot for each cell line was then plotted vs. relative glioma invasiveness. The lines through the data indicate the median for each invasiveness category; *indicates a statistical difference (one-tailed, unpaired t test) between the two categories of invasiveness at p<0.05.

Eighteen of these xenograft lines were used to establish orthotopic xenografts in mice, and mice were observed until reaching a moribund state. The brains were then sectioned and relative invasiveness was determined using H&E staining on up to 10 mice per cell line. These xenograft lines were categorized according to whether they were highly, moderately, minimally, or non invasive (Figure S2). For subsequent analysis the highly and moderately invasive lines were combined into one category containing 8 lines (GBMs 6, 8, 15, 16, 26, 34, 38, and 44) and the minimally and non invasive lines were combined into a second category containing 10 lines (GBMs 5, 10, 12, 14, 22, 28, 36, 43, 46, and 59). The expression of E-cadherin in the corresponding flank lysates was quantified relative to the amount of actin protein in each lysate. This quantification was then plotted versus the relative invasiveness of the xenograft lines in mouse brain (Figure 3). Five of the eight highly/moderately invasive lines expressed substantial amounts of E-cadherin (GBMs 6, 8, 16, 26, 34) while none of the ten minimally/non-invasive lines did. The data revealed that E-cadherin expression was higher in the more invasive xenograft lines, suggesting that E-cadherin expression and/or signaling may contribute to GBM tumor aggressiveness.

### E-cadherin expression in conventional GBM cell lines

**Figure 4:**
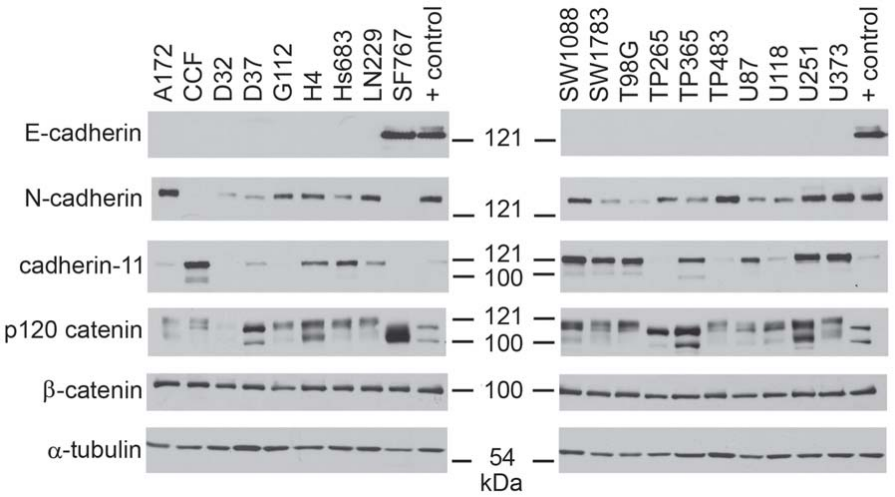
E-cadherin expression in conventional glioma cell lines is rare. Nineteen conventionally grown glioma cell lines were examined by Western Blot for expression of various cadherin and catenin proteins. α-tubulin serves as a loading control. Positive control lysates are as in Figure 2. SF767 was the only conventional glioma cell line examined that expressed E-cadherin.

To further probe the role of E-cadherin in GBMs, we wanted to apply standard cell biology techniques, including using interfering RNA (RNAi) technology to knockdown E-cadherin expression. Because conventionally cultured cell lines are more easily manipulated, a panel of nineteen conventionally cultured glioma cell lines was screened by Western blot for the presence of E-cadherin, N-cadherin, cadherin-11, β-catenin, and p120 catenin (Figure 4). As was seen with the xenograft cultured GBM lines, β-catenin and p120 catenin were expressed in all lines and most lines expressed N-cadherin and/or cadherin-11. Only the SF767 cell line, derived from a recurrent anaplastic astrocytoma [41], was found to express E-cadherin. Importantly, identical results were obtained using the HECD1 anti-E-cadherin antibody (data not shown). SF767 cells did not express either of the other two cadherins evaluated. An original clone of the SF767 cell line was independently obtained from the line's original source (the UCSF/Neurosurgery Tissue Bank) and showed an identical pattern of cadherin expression (Lewis-Tuffin, data not shown). SF767 cells co-segregate with several other GBM cell lines and primary GBM tumors in gene expression studies [42]. Therefore, subsequent experiments probing E-cadherin's role were carried out in the SF767 cell line under conventional culture conditions.

### Role of E-cadherin in SF767 cell growth and motility

**Figure 5:**
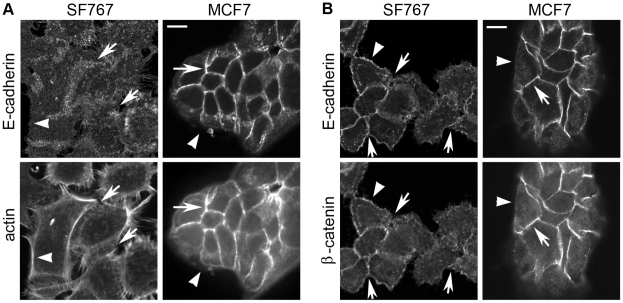
SF767 cells lack junctional organization of actin and have disorganized adhesive structures containing E-cadherin. A. Immunofluorescence for E-cadherin expression and actin localization was carried out on paraformaldehyde-fixed/Triton X-100-permeabilized SF767 and MCF7 cells (as a control). Arrows indicate areas of cell-cell contact; arrowheads point to areas of the plasma membrane without cell-cell contact. MCF7 cells form compact cell-cell adhesions to which the actin cytoskeleton and E-cadherin tightly localize. In contrast, E-cadherin localization in SF767 is at cell-cell contacts and on the plasma membrane at areas without cell-cell contact. Additionally, the actin cytoskeleton is not properly organized at areas of cell-cell contact. 63X magnification. The scale bar is 10 µm and applies to all images in Figure 5. B. Immunofluorescence for E-cadherin and β-catenin expression was carried out on MeOH-fixed/permeabilized SF767 and MCF7 cells. Arrows and arrowheads are as in A. β-catenin and E-cadherin both localize tightly to adherens junctions between MCF7 cells. In contrast, fewer proper cell-cell junctions exist in the SF767 cells. Both E-cadherin and β-catenin localization is diffusely distributed on the plasma membrane, in addition to a disorganized presence at areas of cell contact. 63X magnification.

Initially, the localization of E-cadherin was assessed in SF767 cells using immunofluorescence (Figure 5). When grown under conventional culture conditions, these cells exhibit a “spiky” appearance in which the cells are surrounded by filopodia. This morphology is relatively common in mesenchymal cells but not in cells that express functional, E-cadherin-based cell-cell adhesion complexes, such as MCF7. The difference is particularly evident when the organization of the actin cytoskeleton is examined (Figure 5A). Actin protrusions into filopodia are evident on all sides of the SF767 cells, including between adjacent cells at what should be areas of cell-cell contact. This unusual morphology is in contrast to the very tight areas of cell-cell contact exhibited by the MCF7 cells, in which the actin cytoskeleton is organized in junctional actin rings such that they cannot be distinguished from each other in adjacent cells. Co-staining for E-cadherin in the SF767 cells vs. the MCF7 cells revealed a similarly unusual distribution. In MCF7 cells localization of E-cadherin was restricted to the plasma membrane at areas of cell-cell contact (Figure 5A,B). In SF767 cells E-cadherin was found across the entire surface of the plasma membrane, as well as being distributed in some cytoplasmic locations, particularly in peri-nuclear areas. At areas of what should be cell-cell contact, E-cadherin staining was diffuse and cells exhibited gaps and/or filopodia protrusions.

As a second means of examining areas of cell-cell contact, SF767 and MCF7 cells were co-stained for E-cadherin and β-catenin (Figure 5B). In MCF7 cells E-cadherin and β-catenin had similar distributions to each other and were restricted primarily to intercellular junctions. E-cadherin and β-catenin also co-localized in SF767 cells. However their distribution was not restricted to areas of cell-cell contact, with E-cadherin and β-catenin also found in peri-nuclear areas and along cell edges without adjacent cells. Even at cell-cell contact areas, many of the cells lacked tight junctional staining, and exhibited gaps and/or filopodia protrusions (illustrated for example by the top arrow in Figure 5B). These features, combined with the lack of actin re-organization, suggested the absence of mature adherens junctions. The apparent lack of mature adherens junctions in the SF767 cells raised the question as to whether the E-cadherin expressed in these cells might be mutated. Accordingly, E-cadherin mRNA was isolated from these cells, converted to cDNA, and sequenced. No mutations in the amino acid coding sequence were found (Lewis-Tuffin, data not shown). Taken together, these observations suggest that E-cadherin-mediated, cell contact-dependent signaling may be deregulated in SF767 cells.

**Figure 6:**
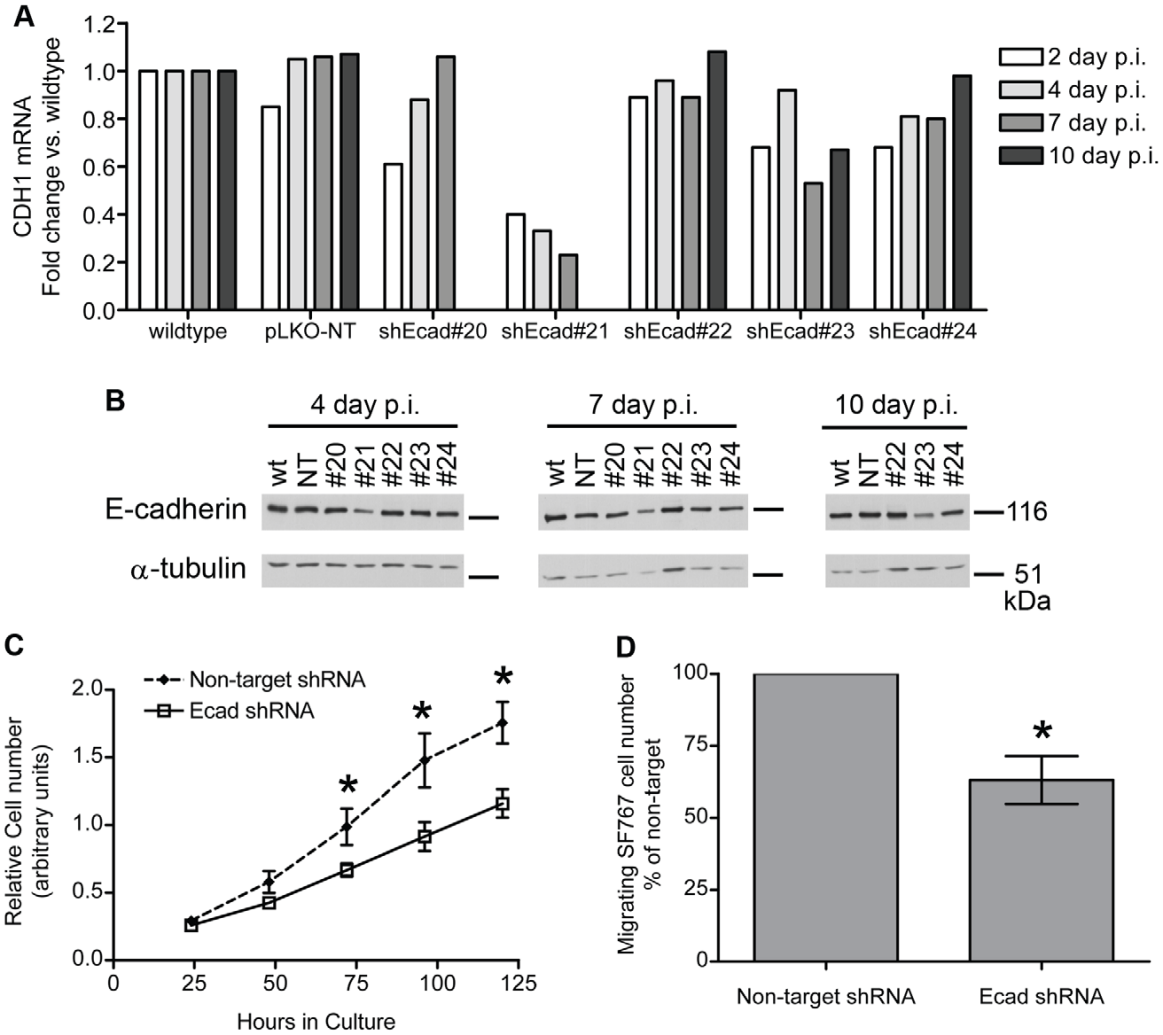
E-cadherin depletion inhibits SF767 growth and migration. A. SF767 cells were infected with lentivirus expressing either non-target shRNA or E-cadherin shRNA on day zero, and then harvested on days 2, 4, 7, and 10 post-infection (p.i.). RNA from the cells was used for quantitative RT-PCR analysis of *CDH1* mRNA, normalized to the expression of *GAPDH* mRNA; data is plotted as fold change vs. the normalized *CDH1* levels in wild-type SF767 cells. The shEcad#20 cell line was discarded after 7 days p.i. due to lack of difference with the pLKO-NT cells in terms of growth rate, morphology, and *CDH1* mRNA expression. The shEcad#21 cell line grew poorly and was completely harvested by 7 days p.i. B. Additional cells were harvested on days 4, 7, and 10 p.i. for protein analysis. Western blot was used to determine knockdown of E-cadherin; α-tubulin is a loading control. The generally poor condition of SF767-shEcad#21 cells by day 7 p.i. is reflected in the reduced levels of α-tubulin seen on the blot, despite loading equal µg of total protein from each sample. C. Growth rates of E-cadherin-depleted SF767-shEcad#23 vs. control SF767-non target cells were determined over 5 days using an MTT assay. E-cadherin-depleted SF767 cells grew more slowly than control SF767 cells. This difference is statistically significant at 72, 96, and 120 hours in culture (2-way ANOVA followed by Bonferroni post hoc tests; * indicates p<0.05). These two cell lines were generated independently of those displayed in parts A and B. D. Migration of E-cadherin-depleted SF767-shEcad#23 vs. control SF767-non target cells (the same cell lines used for the growth experiment in C) was determined using a Boyden chamber trans-well migration assay. Fewer E-cadherin-depleted SF767 cells than control SF767 cells migrated toward the chemoattractant. The difference is statistically significant (paired t-test; * indicates p<0.05).

To begin to address the role of E-cadherin in SF767 growth and migration, a lentiviral-based RNA knockdown approach was used. SF767 cells were infected with lentivirus expressing either non-target or one of five, non-overlapping, E-cadherin targeted shRNAs. Two days later the cells were put into antibiotic selection to produce a polyclonal population of cells with a stable knockdown of E-cadherin. However, despite three attempts, the few cell lines that survived selection contained E-cadherin protein that was either not decreased or only partially affected (Lewis-Tuffin and Huveldt, data not shown). To investigate the absence of good E-cadherin knockdown, we repeated the six infections and examined E-cadherin mRNA and protein levels at 2, 4, 7, and 10 days post infection (Figure 6A,B). The data suggest a direct correlation between E-cadherin expression and SF767 cell growth (Figure 6A, B). Cells infected with the shRNA that produced the strongest depletion of endogenous E-cadherin (shEcad#21) died off gradually but completely within 2 weeks post infection. Cells infected with shRNAs that did not affect E-cadherin levels (non-target shRNA, shEcad#20, or shEcad#22) were viable. Cells infected with shRNAs that resulted in a partial E-cadherin depletion generated polyclonal populations that either lost E-cadherin suppression over time (shEcad#24), or exhibited a partial reduction of E-cadherin levels for the duration of these studies (shEcad#23) (Figure 6A, B). Importantly, the shEcad#21 and shEcad#23 viruses were very effective (80–90% reduction) in reducing endogenous E-cadherin expression in MCF7 cells, without any evidence of a growth deficit in the cadherin-depleted MCF7 cells (Figure S3). Taken together, these results strongly suggest that SF767 cells are dependent on E-cadherin expression for growth.

To follow up on this observation, we further examined the only stable SF767 E-cadherin knockdown line that we obtained, which was an incomplete knockdown produced by the shEcad#23 shRNA. Even this partial E-cadherin knockdown resulted in SF767 cells that grew more slowly in culture than did their control, non-target shRNA counterparts (Figure 6C). We also examined the ability of these E-cadherin depleted cells to migrate towards a gradient of FBS. To control for the growth effects of the E-cadherin reduction, cells in this transwell assay were only allowed to migrate for 6 hours. Figure 6D shows that the migration of E-cadherin-reduced SF767 cells was significantly slower than that of their non-target shRNA counterparts in this assay. Taken together, these results indicate that E-cadherin plays a positive role in the ability of the SF767 glioma cell line to grow and migrate *in vitro*.

## Discussion

E-cadherin is usually assigned a tumor suppressor role in epithelial cells because it is lost in many carcinomas and its loss is associated with a less favorable prognosis [43], [44]. Loss of E-cadherin in these tumors is associated with increased Ras, Rac1, and MAPK signaling, which induce tumor cell growth and invasion [45], [46]. Conversely, re-expression of E-cadherin in such tumor cells suppresses tumor cell growth and invasion [45], [46]. However, under some circumstances, E-cadherin is associated with increased tumorigenesis and tumor dispersion. Indeed, E-cadherin expression, which is absent from the normal, mesenchymally-derived ovary, is upregulated in the vast majority of malignant ovarian tumors and correlates with increased survival, proliferation, and metastatic spread of ovarian cancer cells [47], [48].

Our data, presented here, are consistent with the idea that E-cadherin expression is also an important component of tumor growth and invasiveness in a rare subset of high-grade gliomas. We have shown that, in patients with GBM exhibiting an epithelial/pseudo-epithelial morphology, E-cadherin expression correlates with a worse prognosis compared to patients that did not express E-cadherin. E-cadherin expression also correlates with increased invasiveness of glioma xenograft cell lines in an orthotopic mouse model of invasion. Finally, endogenous E-cadherin expression promotes the growth and migration of the conventional SF767 glioma cell line. Collectively, these results suggest an atypical role for E-cadherin in brain glial tumor biology that, while uncommon, may be of particular importance when it does occur. Our results suggest that the cadherin class of cell adhesion molecules in general, and E-cadherin in particular, may play a key role in the biology of malignant gliomas.

The role and rarity of E-cadherin in gliomas contrasts with the expression of E-cadherin in another type of nervous system tumor: meningiomas. Meningiomas are derived from meningothelial/arachnoid cells, which are some of the few normal nervous system cells that do express E-cadherin. E-cadherin is expressed in most meningiomas [21], [22], [49], and its loss may be associated with tumor progression [20]. In contrast, in the case of GBM, epithelial differentiation with E-cadherin expression is considered a metaplastic (conversion) process in a glial tumor, similar to the more commonly observed malignant mesenchymal component of gliosarcomas. This interpretation is supported by the finding that the glial and metaplastic components in either tumor type (gliosarcoma or GBM) both exhibit similar genetic alterations and apparently arise from a common precursor [33], [50], [51].

Our data on E-cadherin expression is consistent with the majority of previous studies and shows that E-cadherin is not expressed in normal brain, including neuronal cells, astrocytes, oligodendrocytes, or the majority of GBMs. As 18 different classical cadherins are thought to be expressed in brain, cadherin-specific antibodies should be carefully screened for non-specific effects, as indicated by our results with a widely used anti-E-cadherin antibody (clone 36). The lack of E-cadherin expression in normal brain also suggests that a classic EMT is not involved in glioma progression. However, the process of EMT includes the upregulation and downregulation of a variety of genes and proteins, not just cadherins, which together cause the tumorigenic transition [15], [19]. The lack of classic epithelial-to-mesenchymal cadherin-switching in ordinary glial tumorigenesis does not mean that these other aspects of EMT are nonoperational. For example, all of the E-cadherin positive GBMs with epithelial or pseudo-epithelial differentiation were also positive for N-cadherin, a classic EMT marker (Rodriguez, data not shown). In addition, many conventional or serially transplanted GBM cell lines express cadherin 11 (Figure 2, 4), another classic EMT marker. Preliminary data suggest that cadherin 11 can promote GBM cell growth and migration (Lewis-Tuffin, unpublished observations). Indeed, a progression from a pro-neural to a mesenchymal phenotype has been suggested by genomic studies in GBM tumors, and is associated with poor prognosis [52].

E-cadherin expression in GBM appears to be an exception to the EMT rule: when E-cadherin expression is turned on in these tumors it is related to tumorigenesis and poor prognosis. The molecular mechanisms underlying the contribution of E-cadherin to growth and/or invasiveness in GBM are currently unknown. Previous studies in epithelial tumor cells have shown that mesenchymal cadherins (i.e. N-cadherin and cadherin 11) can promote tumor cell growth and migration via p120-mediated activation of Rac1 signaling [45], [46]. Despite an initial activation of Rac1 upon epithelial cell-cell contact, the overall level of Rac1 activity is normally suppressed by E-cadherin expression [45]. However, this E-cadherin effect is thought to be mediated by the formation of mature cell-cell junctions [53], [54]. In the SF767 glioma cell line, mature cell-cell junctions fail to form. It is therefore possible that deregulated E-cadherin signaling causes increased Rac1 activation and induces tumor cell growth and invasion of human glioma cells.

Another possible mechanism by which E-cadherin could regulate proliferation and parenchymal infiltration involves adhesion-induced ligand-independent activation of the EGF receptor, leading to Akt and MAPK activation. The occurrence of such a mechanism has been demonstrated in the OVCAR-3 ovarian cancer cell line and is thought to underlie E-cadherin effects in ovarian cancer in general [47]. This mechanism is particularly intriguing to consider in the context of GBM, which is known for the frequency with which it displays EGFR overexpression or expression of the constitutively-active EGFRvIII mutant. It is of note that GBMs with epithelial and “adenoid” morphology show a decreased frequency of EGFR amplification [33]. E-cadherin expression could represent an alternative mechanism for activating the EGF pathway in this subset of tumors.

Finally, E-cadherin expression could contribute to the migration mechanics used by the invading tumor cells. Gliomas are known for their diffusely infiltrative invasion pattern, in which the tumor cells migrate as single cells. However the heterogeneity of glioma genotypes, phenotypes, and surrounding tissue architecture suggest that a diversity of migration mechanisms could be used, even within the same tumor, at different stages of tumor dispersal. Although E-cadherin expression in gliomas is rare, when it occurs it could facilitate collective tumor cell migration strategies, including migration as discrete cell clusters or as multicellular strands or sheets, which are known to be dependent on cadherin expression [55].

One question is whether the pro-tumorigenic function of E-cadherin applies widely in gliomas or narrowly to GBM tumors with pseudo-epithelial differentiation. Arguing for the latter, ectopic overexpression of E-cadherin in the U87 glioma cell line did not increase, but rather slightly decreased migration *in vitro* (Lewis-Tuffin, unpublished observations). Unlike SF767 cells, which only express E-cadherin, U87 cells also express endogenous N-cadherin and cadherin-11, which could also affect migration- and proliferation-related signaling. Therefore predicting the effect of ectopic E-cadherin in GBM cell culture is not straightforward. This will be an important issue to explore in future studies.

In conclusion, we have identified an unexpected association of E-cadherin expression with aggressive biologic behavior in a rare subset of GBM. The association of E-cadherin expression with adverse clinical behavior should be interpreted with caution, given the retrospective nature of the clinical/therapeutic data collected. Nonetheless, both our clinical and pre-clinical data support the conclusion that E-cadherin expression is associated with increased aggressiveness in human GBM. These findings provide a glimpse into the importance of the cadherin class of cell-cell adhesion molecules in the biology of high grade astrocytic tumors. Future studies should functionally dissect the specific molecular mechanisms involving cadherins and associated proteins in glioma biology. This will facilitate the identification of more reliable prognostic biomarkers, and perhaps the development of novel therapies.

Finally, our findings may also be relevant to epithelial tumor biology. Despite the significance of EMT in epithelial tumor progression and dissemination, many epithelial metastases retain E-cadherin expression. The possibility that deregulated E-cadherin function may promote the aggressiveness of these tumors warrants further investigation.

## Materials and Methods

### Ethics Statement

All patient-related studies were approved by the Mayo Clinic Institutional Review Board and therefore have been performed in accordance with the ethical standards laid down in the Declaration of Helsinki, including obtaining written informed consent prior to donation of tissue. Animal studies were approved by the Mayo Clinic Institutional Animal Care and Use Committee (IACUC A5409) and were conducted according to Mayo Clinic IACUC guidelines for animal husbandry.

### GBM tissue microarray tumor specimens and immunohistochemical analysis

Construction of the two Mayo Clinic human tissue microarrays was performed as described in a previous publication [33]. Briefly, 83 cases of human high grade astrocytomas (11 cases of grade III astrocytomas, 72 cases of GBMs) were used to construct two tissue microarrays consisting of 3–4 0.6 cm diameter cores per tumor. Each tissue microarray also contained samples of non-neoplastic gray and white brain matter (obtained from epileptigenic brain), infiltrating ductal carcinoma breast tissue, six GBM xenograft cell lines, and U87 and U251 conventional glioma cell lines. The human glioma rim-core tissue microarray was a gift from Michael E. Berens (Translational Genomics Research Institute, Phoenix, AZ) and has been described previously [34]. Immunohistochemistry for E-cadherin was performed on the three tissue microarrays as described previously [33] using a monoclonal human anti E-cadherin antibody (clone 4A2C7, Zymed/Invitrogen, Carlsbad, CA).

### Tumor specimens and immunohistochemical analyses of GBMs with epithelial/pseudo-epithelial differentiation

Tumor classifications, tissue microarray construction, immunohistochemistry to analyze E-cadherin expression, and immunohistochemical scoring were described in detail in a previous publication [33]. Briefly: tumors from 32 patients from a previously characterized retrospective series of glioblastomas with varying degrees of epithelial morphology were stained with a monoclonal human anti E-cadherin antibody (clone 4A2C7, Zymed/Invitrogen, Carlsbad, CA) using tissue microarray sections. These tumors represent a rare subtype of GBM (<2%) and were largely derived from pathology consultations. Clinical follow-up was necessarily limited, but present in 27 patients (84%): 7 women and 20 men. The median age at diagnosis was 56 years (interquartile range 44–67). The tumors were histologically classified by previously published criteria as adenoid-GBM (n = 11), epithelioid GBM (n = 4) or GBM with true epithelial differentiation (n = 12). Postoperative treatment approaches included radiation therapy to the brain (50–60 Gy)(n = 15), with chemotherapy (n = 6), including Temodar in 5 patients, or precise treatment modality unknown (n = 12). Immunohistochemical scoring as either positive or negative was performed by a single, blinded neuropathologist (F.J.R.). Univariate analyses of E-cadherin immunoexpression with respect to overall and recurrence-free survival, as well as relevant clinicopathologic features were performed using the log-rank test, and illustrated with Kaplan Meier curves. All analyses were 2-sided with p-values <0.05 considered statistically significant.

### Xenograft information

Each of the 19 serially passaged xenograft cell lines used in this study are derived from resected tumors from different human patients and are propagated by serial transplantation in the flank of nude mice [38]. Tumors are propagated exclusively *in vivo* in order to preserve molecular and histopathologic features of the primary patient tumor specimens. Eighteen of the xenograft cell lines have been described previously [38], [56]–[58]. GBM59 has not been reported previously; the original tumor from which GBM59 was derived was also diagnosed as a GBM (Carlson and Sarkaria, unpublished manuscript).

### Orthotopic xenograft model and evalution of relative invasiveness

Short-term explant cultures derived from flank tumor xenografts were injected orthotopically into nude mouse brain as described previously [58]. Mice were euthanized when they reached a moribund condition. Brains from these mice were resected and then bisected along the needle tract used for injecting the tumor cells. Half of each bisected brain was placed in formalin and subsequently embedded in paraffin. These tissue samples were processed for routine H&E staining in the TACMA Mayo core laboratory. All injected tumors formed a dominant mass of several millimeters in the cerebral hemisphere ipsilateral to the injection, up to a maximum diameter of approximately 6 mm in the moribund state. The degree of invasiveness was evaluated on the H&E sections by an observer (C.G.) who was blind to tumor E-cadherin status. In tumors which were classified as “highly invasive”, tumor cells could be easily identified extending along the commissural structures (corpus callosum and anterior commissure) to the opposite hemisphere. In tumors which were considered “minimally invasive” or “non invasive” a “nearly sharp” border could be identified between the tumor mass and surrounding brain parenchyma, with minimal or no intermixing of tumor cells with surrounding parenchyma at the edge of the tumor (Figure S2). A number of xenografts showed intermediate features (“moderate invasiveness”) with cells extending well away from the main tumor mass, but largely remaining in the ipsilateral hemisphere.

### Conventional cell culture

Established conventional cell lines were cultured on tissue-culture treated plastic dishes at 37°C, 5% CO_2_, in DMEM media containing 10% Fetal Bovine Serum (not heat-inactivated), an additional 2 mM L-glutamine, and 1% non-essential amino acids. Glioma cell lines include A172, CCF, D32, D37, H4, Hs683, SW1088, SW1783, TP265, TP365, TP483, U87, U251, and U373 (obtained from Bob Jenkins, Mayo Clinic, Rochester, MN); G112P, SF767, T98G, and U118 (obtained from Joe Loftus, Mayo Clinic, Scottsdale, AZ); LN229 (obtained from Wei Zhang, MD Anderson Cancer Center, Houston, TX); and SF767 (obtained from the UCSF/Neurosurgery Tissue Bank, San Francisco, CA). MCF7, MDA-MB-231, and UMRC3 cell lines were also used.

### Constructs

The MISSION Non-Target shRNA control vector pLKO-non target (SHC002) and pLenti-human E-cadherin shRNA vectors, both expressing a puromycin resistance gene, were purchased from the Mission RNAi Consortium shRNA collection (Sigma-Aldrich, St. Louis, MO) and obtained from the Mayo Clinic Comprehensive Cancer Center RNA Interference Techonology Resource. E-cadherin shRNA product numbers are: pLKO-shEcad#20, TRCN0000039663; pLKO-shEcad#21, TRCN0000039664; pLKO-shEcad#22, TRCN0000039665; pLKO-shEcad#23, TRCN0000039666; pLKO-shEcad#24, TRCN0000039667.

### Virus production and infections

Lentivirus stocks were produced using Virapower™ lentivirus packaging mix and the 293FT cell line according to the manufacturer's protocol (Invitrogen, Carlsbad, CA). SF767 cells (obtained from J. Loftus) grown to 50% confluence were incubated for 24 hours in a 1∶1 dilution of virus:media with 4 µg/ml Polybrene. After a 24-h recovery in complete media without virus, polyclonal stable cell lines were selected and maintained in media containing 5 µg/ml puromycin. In the parallel SF767 and MCF7 infection experiment, a 1∶4 dilution of virus:media with 6 µg/ml Polybrene was used.

### Preparation of whole cell lysates for western blot

Flank xenograft tissue lysates: Flash frozen flank xenograft tissues were homogenized at 0°C in SDS Lysis buffer (2% w/v SDS, 4 M deionized urea, 62.5 mM Tris-Cl pH 6.8, 1 mM EDTA, 5% v/v β-Mercaptoethanol, with 1 mM Na_3_VO_4_, 50 mM NaF, and 1 mM PMSF). Lysates were then cleared by centrifugation at 13000 rpm at 4°C for 20 minutes. Total protein in the supernatents was quantified using the nitric acid method [59], while the remaining lysates were mixed with Laemmli Sample Buffer (2X final concentration, 0.1 M Tris-Cl pH 6.8, 2% SDS, 10% sucrose, 0.24 M β-Mercaptoethanol, 0.008% bromophenol blue) and boiled for 5 minutes before being analyzed by Western Blot.

Conventional cell culture lysates: Whole cell lysates of conventionally cultured cell lines were made by lysing the cells in ice cold RIPA buffer (50 mM Tris-Cl pH 7.4, 150 mM NaCl, 1% Igepal CA-630 (NP-40 substitute), 0.5% Deoxycholic Acid, 0.1% SDS, with 1 mM Na_3_VO_4_, 1 mM EDTA, 50 mM NaF, 1 mM PMSF, 5 µg/ml leupeptin, and 2 µg/ml aprotinin) for 7 minutes, followed by brief sonication. Ten µl samples of each lysate were quantitated for total protein using the BioRad Protein Assay Dye Reagent (BioRad Laboratories, Hercules, CA). Remaining lysates were mixed with Laemmli Sample Buffer (2X final concentration) and boiled for 5 minutes before being analyzed by Western Blot.

### Western blotting

Equal µg amounts of protein lysates were separated by SDS-PAGE and transferred to nitrocellulose filters using standard methods. Blots were blocked in 5% nonfat dry milk in Tris-buffered saline (TBS), pH 7.4 before being incubated in primary antibodies in 5% milk/TBS. Blots were rinsed three times and washed four times 5 minutes in TBS +0.1% Tween 20 (TBST). Blots were then incubated with HRP-conjugated secondary antibodies in 5% milk/TBS, and rinsed and washed in TBST as before. Proteins were detected using Amersham ECL Western Blotting Detection Reagents (GE Healthcare, Buckinghamshire, United Kingdom). Primary antibodies were as follows: mouse anti-p120 catenin (clone 15D2, Zymed/Invitrogen), rabbit anti-β catenin (C2206, Sigma-Aldrich), mouse anti-E-cadherin (clone 36, BD Biosciences, San Jose, CA), mouse anti-N-cadherin (clone 3B9, Zymed/Invitrogen), mouse anti-cadherin-11 (clone 5B2H5, Zymed/Invitrogen), rabbit anti-actin (A2066, Sigma-Aldrich), and mouse anti-α-tubulin (clone B-5-1-2, T5168, Sigma-Aldrich). HRP-conjugated secondary antibodies (anti-mouse and anti-rabbit) were obtained from Jackson ImmunoResearch Laboratories, West Grove, PA.

### Immunofluorescence

SF767 and MCF7 cells were plated on glass coverslips and allowed to adhere for a minimum of 24 hours. For combined E-cadherin and actin analysis, cells were fixed with 3% paraformaldehyde at room temperature for 30 minutes, then washed twice in PBS+10 mM glycine. Cells were permeabilized with PBS/0.2% Triton X-100 for 2.5 minutes at room temperature, then washed again with PBS/glycine before blocking and applying mouse anti-E-cadherin primary antibody (clone 36, BD Biosciences). For combined E-cadherin and β-catenin analysis, cells were fixed/permeabilized by incubation in 100% methanol at −20°C for 7 minutes, then washed with PBS before blocking and applying mouse anti-E-cadherin and rabbit anti-β catenin (C2206, Sigma-Aldrich) primary antibodies. Secondary antibodies were goat anti mouse Alexa 488 and goat anti rabbit Alexa 594 (Invitrogen). Actin was localized by incubation with Alexa 594-conjugated phalloidin (Invitrogen). SF767 and MCF7 cells were stained simultaneously using identical conditions. Coverslips were mounted on glass slides with Aqua Poly/Mount (Polysciences, Inc. Warrington, PA). Cells were visualized with a Zeiss LSM 510 META laser scanning confocal microscope (Carl Zeiss Microimaging, Heidelberg, Germany) using a Plan-Apochromat 63x/1.4 oil immersion objective. Images were acquired with the Zeiss AIM LSM510 software using a scan zoom of 1.7 and compiled in Adobe Photoshop.

### Quantitative RT-PCR analysis

Cells infected with non-target or shEcad-expressing viruses were rinsed with 1xPBS. A portion of the cells on each plate were scraped up using a cell lifter and transferred in PBS to a sterile microfuge tube. The remaining cells were returned to culture conditions for future sampling. For harvests in which both protein and RNA were to be isolated, the cell sample was divided in half prior to collecting the cells by centrifugation; the individual cell pellets were then lysed for either RNA or protein isolation. Total RNA was isolated using the miRCURY RNA Isolation Kit - Cell and Plant (Exiqon Inc., Woburn, MA) according to manufacturer protocol. Equal ng amounts of total RNA were converted to cDNA using the High Capacity cDNA Reverse Transcriptase Kit (Applied Biosystems Inc., Foster City, CA). qPCR reactions were performed in triplicate with 10 ng of cDNA and TaqMan® FAST Universal PCR master mix (Applied Biosystems). Human CDH1 (ABI# Hs00170423_m1) and human GAPDH (ABI# Hs99999905_m1) primer/probe sets were purchased from Applied Biosystems. All amplification data were collected with an Applied Biosystems Prism 7900 sequence detector and analyzed with Sequence Detection System software (Applied Biosystems). Data were normalized to GAPDH, and mRNA abundance was calculated using the ΔΔ*C*_T_ method [60]. Samples with an average GAPDH *C*_T_ value greater than 1 *C*_T_ (1 fold-change) away from the wild-type GAPDH *C*_T_ value were excluded from further analysis.

### Boyden chamber migration assay

Migration assays were performed on 10 mm polycarbonate membrane, 8 µm pore size, transwell culture inserts (upper chamber) (Nalge Nunc International, Rochester, NY) coated with 15 µg/ml Bovine Type I collagen (BD Biosciences) and placed in 24 well plates (lower chamber). The day before the assay, culture medium on the cells was changed to DMEM containing 250 µg/ml BSA, with additional 2 mM L-glutamine and 1% non-essential amino acids, and the cells incubated overnight at 37°C, 5% CO_2_. The day of the assay, cells were harvested by a very brief incubation in 0.25% Trypsin-EDTA, counted using Trypan Blue exclusion to determine the number of live cells, and resuspended in DMEM/BSA at 5×10^5^ cells/ml. 1×10^5^ cells were placed in the upper chamber; DMEM/BSA containing 0.5% Fetal Bovine Serum was placed in the lower chamber. Cells were allowed to migrate to the underside of the transwell for 6 hours at 37°C, 5% CO_2_. Any cells that remained in the upper chamber were removed by gently scrubbing with cotton swabs. Cells on the underside of the transwell chambers were collected by incubating the bottom of the chamber in 225 µl of Cell Dissociation Buffer (equal parts 0.5% phenol red-free Trypsin EDTA (Invitrogen) and Hank's Balanced Salt Solution (5.37 mM KCl, 0.45 mM KH_2_PO_4_, 137 mM NaCl, 4.17 mM NaHCO_3_, 0.34 mM Na_2_HPO_4_, 5.55 mM D-glucose)) for 20 minutes at 37°C, 5% CO_2_. At the end of this time 75 µl of a 4x Lysis+Dye Solution (20x Lysis Solution and 400x GR DNA Binding Dye, both from the CyQuant Cell Proliferation Assay kit (Invitrogen), diluted in water to make a 4x stock solution) was added to the cell-containing samples in the lower chamber under each transwell. Samples were frozen at −80°C for at least 1 hour. They were then transferred to a black, clear-bottom, 96-well plate and sample fluorescence read by a SpectraMax M5 plate reader running SOFTmax PRO software (Molecular Devices Corporation, Sunnyvale, CA) with excitation at 480 nm and absorption at 520 nm. Sample fluorescence for each cell type was normalized to the fluorescence obtained from 0.25×10^5^ cells plated directly in triplicate wells of a 24 well plate, allowed to adhere for 6 hours at 37°C, 5% CO_2_, and then quantified by the same method (incubation with Cell Dissociation Buffer and 4x Lysis+Dye Solution followed by one round of freeze-thaw lysis). This quantification method measures DNA concentration to determine the relative number of cells in each sample. Data were expressed as percentage of control (non-target shRNA-transduced) cell migration and are presented as the mean +/− SEM of 4 independent experiments performed in triplicate.

### Cell growth assay

MTT cell growth assays were carried out by plating 4000 cells/well in 96 well plates, with triplicate wells for each harvest. Cells were allowed to grow for 24, 48, 72, 96, or 120 hours. Prior to each harvest, cells were incubated with 1 mg/ml MTT (Sigma-Aldrich) at 37°C, 5% CO_2_ for 1 hour. Formazan precipitate (produced in proportion to the number of live cells) was then extracted from the cells by replacing the media on the cells with 200 µ/well DMSO (Sigma-Aldrich), pipetting up/down, and incubating at 37°C, 5% CO_2_ for 5 minutes. The absorbance at 550 nm was then determined for each sample. Data were plotted as mean +/− SD. This experiment was performed three times in triplicate; a representative plot is presented.

### Statistics

Overall and recurrence-free survivals were evaluated using the Kaplan-Meier method, performed with SAS software (SAS Institute Inc., Cary, NC). Unpaired, one-tail t test (Figure 3), two-way ANOVA and Bonferroni post hoc testing (Figure 6C), and the paired t test (Figure 6D) were done using GraphPad Prism 4 software (GraphPad Software Inc., La Jolla, CA).

## Supporting Information

Figure S1 Comparison of anti-human E-cadherin antibodies for use with immunohistochemistry. Immunohistochemistry for E-cadherin expression was carried out using either the 4A2C7 (Zymed/Invitrogen) or clone 36 (BD Transduction Labs) antibodies on the Mayo Clinic GBM-A3 TMA. Among other samples, this TMA includes non-neoplastic brain tissue (both gray and white matter), a sample of infiltrating ductal carcinoma (IDC) breast tumor, and a plug of U251 glioma cells. The scale bar is 20 µm and applies to all images.
(3.49 MB TIF)

Figure S2 Examples of minimally versus highly invasive orthotopic GBM xenografts. H&E stain of mouse brain orthotopically implanted with GBM xenograft line 12 (minimally invasive) or GBM line 8 (highly invasive). Left images (12.5x magnification) show a section of the entire brain; right images (200x) are magnified to demonstrate the tumor/normal brain interface of the two GBM lines. Left image scale bars are 1 mm; right image scale bars are 50 µm.
(4.05 MB TIF)

Figure S3 Comparison of E-cadherin shRNA effects on SF767 and MCF7 cells. A. SF767 cells were infected with freshly prepared, high titer virus expressing non-target shRNA or shEcad#21, #22, or #23. CDH1 mRNA levels in these cells were quantified using qPCR at days 2, 4, and 6 post infection (p.i.; top); E-cadherin protein expression was determined at days 4 and 6 p.i. using Western blot (bottom). The 6 day p.i. shEcad#21 and 4 and 6 day p.i. shEcad#23 samples are not shown because they did not meet our qPCR normalization criteria. The generally poor condition of the cells on these days is also reflected in the reduced levels of α-tubulin seen on the corresponding Western blots, despite loading equal µg of total protein from each sample. B. MCF7 cells were infected with the same freshly prepared, high titer virus expressing non-target shRNA or shEcad#21, #22, or #23. CDH1 mRNA levels in these cells were quantified using qPCR at days 2, 4, 7, and 15 post infection (top); E-cadherin protein expression was determined at days 4, 7, and 15 p.i. using Western blot (bottom).
(0.97 MB TIF)
