# Supplementary figures and images for: Misregulated E-Cadherin Expression Associated with an Aggressive Brain Tumor Phenotype
Source: PLoS One. 2010 Oct 27;5(10):e13665. doi: 10.1371/journal.pone.0013665 (PMC2965143; doi:10.1371/journal.pone.0013665)

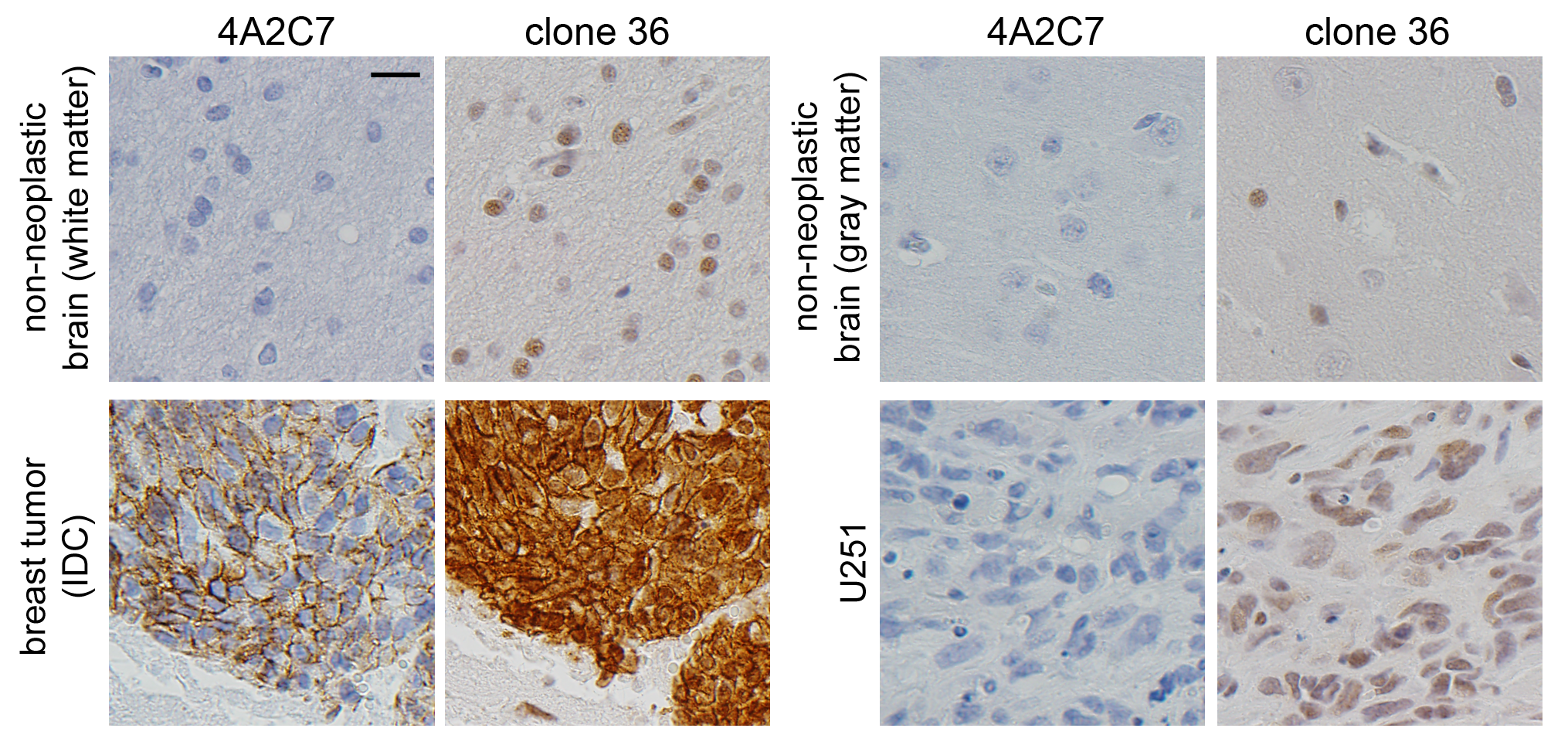

Supplement: Figure S1 — Comparison of anti-human E-cadherin antibodies for use with immunohistochemistry. Immunohistochemistry for E-cadherin expression was carried out using either the 4A2C7 (Zymed/Invitrogen) or clone 36 (BD Transduction Labs) antibodies on the Mayo Clinic GBM-A3 TMA. Among other samples, this TMA includes non-neoplastic brain tissue (both gray and white matter), a sample of infiltrating ductal carcinoma (IDC) breast tumor, and a plug of U251 glioma cells. The scale bar is 20 µm and applies to all images. (3.49 MB TIF) [file pone.0013665.s001.tif]

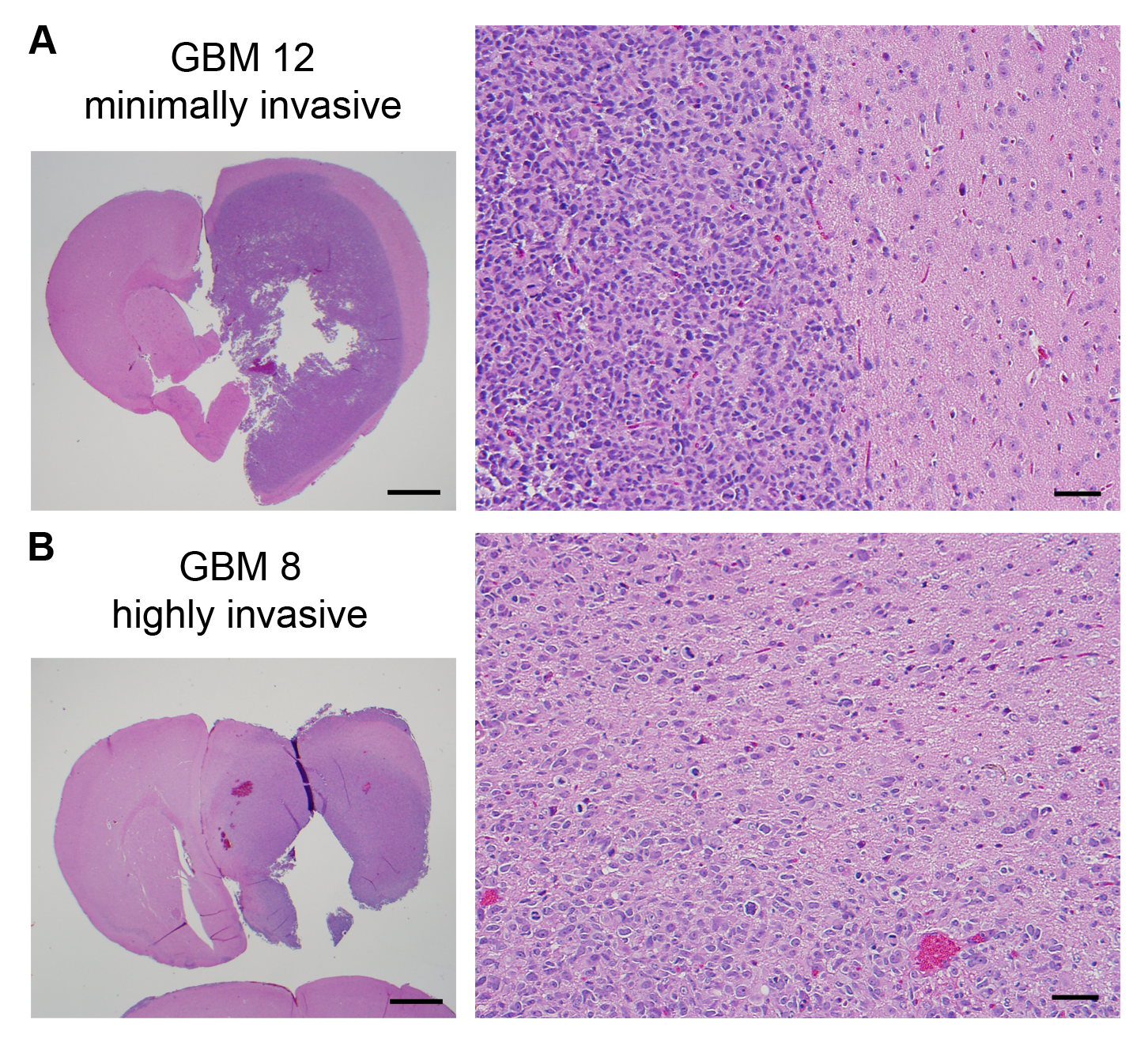

Supplement: Figure S2 — Examples of minimally versus highly invasive orthotopic GBM xenografts. H&E stain of mouse brain orthotopically implanted with GBM xenograft line 12 (minimally invasive) or GBM line 8 (highly invasive). Left images (12.5x magnification) show a section of the entire brain; right images (200x) are magnified to demonstrate the tumor/normal brain interface of the two GBM lines. Left image scale bars are 1 mm; right image scale bars are 50 µm. (4.05 MB TIF) [file pone.0013665.s002.tif]

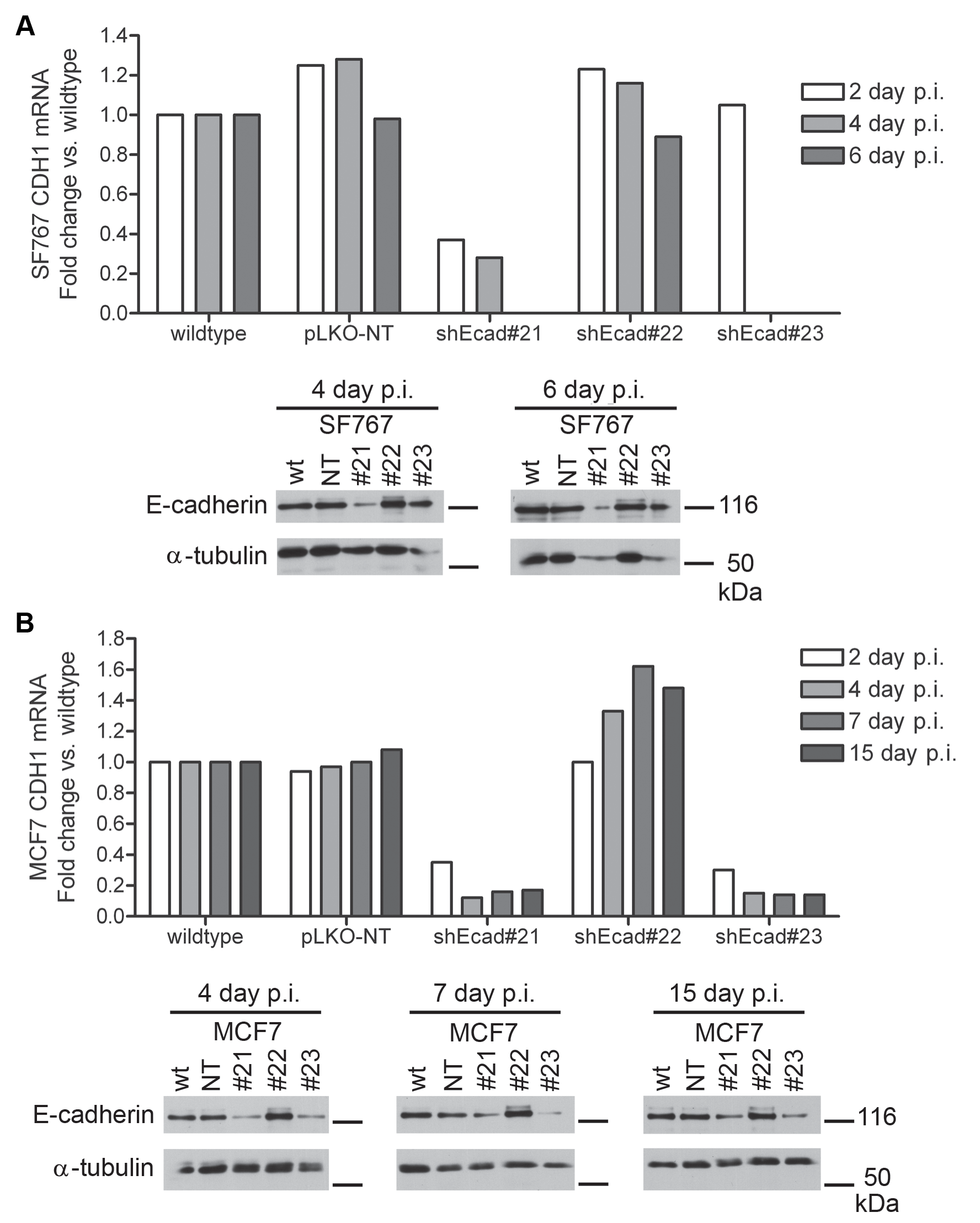

Supplement: Figure S3 — Comparison of E-cadherin shRNA effects on SF767 and MCF7 cells. A. SF767 cells were infected with freshly prepared, high titer virus expressing non-target shRNA or shEcad#21, #22, or #23. CDH1 mRNA levels in these cells were quantified using qPCR at days 2, 4, and 6 post infection (p.i.; top); E-cadherin protein expression was determined at days 4 and 6 p.i. using Western blot (bottom). The 6 day p.i. shEcad#21 and 4 and 6 day p.i. shEcad#23 samples are not shown because they did not meet our qPCR normalization criteria. The generally poor condition of the cells on these days is also reflected in the reduced levels of α-tubulin seen on the corresponding Western blots, despite loading equal µg of total protein from each sample. B. MCF7 cells were infected with the same freshly prepared, high titer virus expressing non-target shRNA or shEcad#21, #22, or #23. CDH1 mRNA levels in these cells were quantified using qPCR at days 2, 4, 7, and 15 post infection (top); E-cadherin protein expression was determined at days 4, 7, and 15 p.i. using Western blot (bottom). (0.97 MB TIF) [file pone.0013665.s003.tif]
